# Deep sequencing reveals important roles of microRNAs in response to drought and salinity stress in cotton

**DOI:** 10.1093/jxb/eru437

**Published:** 2014-11-04

**Authors:** Fuliang Xie, Qinglian Wang, Runrun Sun, Baohong Zhang

**Affiliations:** ^1^Department of Biology, East Carolina University, Greenville, NC 27858, USA; ^2^Henan Institute of Sciences and Technology, Xinxiang, Henan 453003, PR China

**Keywords:** Cotton, deep sequencing, drought, fibre, literature mining, microRNA, salinity.

## Abstract

Drought and salinity are two major environmental factors adversely affecting plant growth and productivity. However, the regulatory mechanism is unknown. In this study, the potential roles of small regulatory microRNAs (miRNAs) in cotton response to those stresses were investigated. Using next-generation deep sequencing, a total of 337 miRNAs with precursors were identified, comprising 289 known miRNAs and 48 novel miRNAs. Of these miRNAs, 155 miRNAs were expressed differentially. Target prediction, Gene Ontology (GO)-based functional classification, and Kyoto Encyclopedia of Genes and Genomes (KEGG)-based functional enrichment show that these miRNAs might play roles in response to salinity and drought stresses through targeting a series of stress-related genes. Degradome sequencing analysis showed that at least 55 predicted target genes were further validated to be regulated by 60 miRNAs. CitationRank-based literature mining was employed to determinhe the importance of genes related to drought and salinity stress. The NAC, MYB, and MAPK families were ranked top under the context of drought and salinity, indicating their important roles for the plant to combat drought and salinity stress. According to target prediction, a series of cotton miRNAs are associated with these top-ranked genes, including miR164, miR172, miR396, miR1520, miR6158, ghr-n24, ghr-n56, and ghr-n59. Interestingly, 163 cotton miRNAs were also identified to target 210 genes that are important in fibre development. These results will contribute to cotton stress-resistant breeding as well as understanding fibre development.

## Highlight

A total of 155 drought/salinity-responsive miRNAs were identified in cotton; many of them target important stress-related genes, including *APS*, *SOD*, *NAC*, *MYB*, and *MAPK*, that are regulated upon abiotic stress.

## Introduction

Drought and high salinity are two of the most severe and wide-ranging abiotic stress factors that inhibit plant growth and development and ultimately negatively affect plant yield or quality (Krasensky and Jonak, 2012). During the long-term evolutionary process, plants have developed a series of regulatory mechanisms to cope with these unfavourable conditions at different levels, including cellular, physiological, biochemical, and molecular processes (Covarrubias and Reyes, 2010). A great deal of effort has been put into identify factors involved in the response to drought and salinity stresses. For instance, it is well established that hormone-mediated signalling cross-talk in plants participates in the response against drought and salinity stress, such as abscisic acid (ABA), salicylic acid, and ethylene (Huang *et al.*, 2012). These studies suggested that gene expression regulation is a crucial strategy for plants to combat drought and salinity stress at the post-transcriptional level (Covarrubias and Reyes, 2010). One of the most important players for gene expression regulation is microRNAs (miRNAs), a class of non-coding small RNA molecules of ~21 nucleotides in length. miRNAs are well known to modulate negatively the expression of their targets either by mRNA cleavage or by translation inhibition, based on perfect or near-perfect complementary nucleotide binding to their target mRNAs (Ambros, 2004). As well as their roles in development and metabolism, a series of miRNAs have been shown to participate in the abiotic and biotic stress response (Dugas and Bartel, 2004; Zhang and Wang, 2015). For example, miR394 is a conserved miRNA that has been identified in a series of plant species, such as *Arabidopsis* (Jones-Rhoades and Bartel, 2004), rice, cotton (Zhang *et al.*, 2007), switchgrass (Xie *et al.*, 2014) and *Brassica napus* (Zhao *et al.*, 2012). Recent studies indicated that miR394 is a versatile miRNA that is involved in multiple stress responses. miR394 was found to be up-regulated by sulphate, cadmium, and iron deficiency (Huang *et al.*, 2010). *Arabidopsis* miR398 was identified to detoxify superoxide radicals by directing the cleavage of its two targets, Cu/Zn superoxide dismutases (SODs; cytosolic CSD1 and chloroplastic CSD2) (Sunkar *et al.*, 2006). Currently, there are estimated to be up to 40 plant miRNA families related to abiotic stress, many of which are associated with salt and drought stress response (Covarrubias and Reyes, 2010; Sunkar, 2010; Wang *et al.*, 2013).

Cotton is one of the leading economic crops in the world mainly because of its natural lint fibre, an important material for clothing, fine paper, and other purposes. Currently, increasing research is being carried out on cotton to improve its fibre yields and quality, including the mechanism of cotton fibre development and cotton’s environmental adaption to salinity and drought (Trivedi *et al.*, 2012; Lu *et al.*, 2013; Zhao *et al.*, 2013). Although cotton is a relatively drought-tolerant and salt-tolerant crop, exposure of cotton to high salinity and excessive water deficit could lead to a series of metabolic disorders in terms of osmotic effects (dehydration), nutritional imbalance, and toxicity of ions, which have a considerable negative impact on cotton growth and lint yield, especially at critical growth stages (Mahajan and Tuteja, 2005; Dong, 2012). To date, based on transcriptome and transgenic analysis, a large number of cotton genes have been shown to display aberrant expression in response to salinity and drought stress either in cotton or in other species. For instance, overexpression of the cotton CBL-interacting protein kinase gene (GhCIPK6) in transgenic *Arabidopsis* resulted in improved tolerance to salt, drought, and ABA stress, indicating that GhCIPK6 might be a positive regulator to fight salt and drought stress in cotton (He *et al.*, 2013). Transgenic tobacco overexpressing the cotton group C mitogen-activated protein kinase (MAPK) gene (GhMPK2) had a lower rate of water loss and exhibited enhanced tolerance to salt and drought, indicating that GhMPK2 might positively regulate salt and drought tolerance in tobacco and cotton (Zhang *et al.*, 2011). Microarray-based transcriptome analysis uncovered some salt/drought-mediated signal transduction pathways in cotton, where a number of candidate genes are expressed differentially and might be potential markers of tolerance to salt and drought stress, such as WRKY, ERF, transmembrane nitrate transporter, pyruvate decarboxylase, and sucrose synthase (Yao *et al.*, 2011; Ranjan *et al.*, 2012). However, the mechanism controlling cotton response to abiotic stress is still unclear. although a great deal of progress has recently been made on cotton genome sequencing. Therefore, identification of salt-responsive and drought-responsive genes in cotton lags behind that in other model species, such as *Arabidopsis* and rice. Also, the regulatory mechanism mediated by these responsive genes is still poorly understood.

MicroRNAs (miRNAs) may play a role during cotton response to drought and salinity stress. Using both computational and deep sequencing technology, some conserved and new miRNAs have recently been identified in cotton (Qiu *et al.*, 2007; Zhang *et al.*, 2007; Pang *et al.*, 2009; Chen *et al.*, 2013; Gong *et al.*, 2013; Yang *et al.*, 2013; Zhang *et al.*, 2013). Some of these miRNAs and their predicted targets were also found to be expressed differentially in terms of dose dependence and tissue dependence under salinity and drought conditions, such as miR156-SPL2, miR162-DCL1, miR159-TCP3, miR395-APS1, and miR396-GRF1 (Yin *et al.*, 2012; Wang *et al.*, 2013). These findings suggest that cotton miRNAs play important roles in response to salt and drought stress. Understanding how miRNAs participate in gene regulation in salt and drought stress could allow improvement of cotton tolerance and adaptation to salt and drought, further resulting in improving fibre yields and quality. However, identification of cotton miRNAs is also hindered, and there has been no genome-wide identification and functional analysis of cotton miRNAs during exposure to drought and salinity stress. Cotton miRNAs are largely unknown, particularly in terms of their function. In this study, three cotton seedling small RNA libraries: control, salt treated and drought-treated, were sequenced. A total of 267 conserved miRNAs and 75 novel miRNAs were identified from the three libraries, in which at least 18 and 27 miRNAs were salt specific and drought specific, respectively. Evidence from miRNA target prediction, Gene Ontology (GO) term classification, Kyoto Encyclopedia of Genes and Genomes (KEGG)-based pathway analysis, and literature-based text mining also support that these identified miRNAs play a critical role in response to salt and drought stress in cotton.

## Materials and methods

### Small RNA library preparation and sequencing

Seeds of *Gossypium hirsutum* L. cultivar TM-1 were sterilized with 70% (v/v) ethanol for 60 s, 6% (v/v) bleach for 6–8min, and then were washed with sterilized water at least four times. The sterilized seeds were germinated on half-strength Murashige and Skoog (MS) medium (pH 5.8) containing 0.8% agar under a 16h light/8h dark cycle at room temperature for 10 d. The MS medium was supplemented with 0.5% NaCl as salinity treatment and with 5% polyethylene glycol (PEG) as drought treatment. Each treatment was replicated five times in five individual culture chambers, and each chamber contained five seeds. Ten-day-old cotton seedlings (controls, 0.5% NaCl, and 5% PEG treatment) were harvested and immediately frozen in liquid nitrogen. Total RNAs was extracted from each tissue sample using the mirVana miRNA isolation kit (Ambion, Austin, TX, USA) according to the manufacturer’s protocol. RNAs were quantified and qualified by Nanodrop ND-1000 (Nanodrop Technologies, Wilmington, DE, USA). All RNA samples were submitted to BGI (Shenzhen, China) for high-throughput sequencing using an Illumina HiSeq high-throughput sequencing platform.

### Pipeline of bioinformatics analysis

All the raw sequences generated from the three small RNA libraries were first cleaned, including removing 5′ and 3′ adaptors and filtering low-quality reads. Then, the raw sequences were categorized into unique reads, and read counts were also calculated. To evaluate the similarity coefficient of the three sequencing libraries, the top 5000 abundant small RNAs were chosen to compute the Jaccard index (Mohorianu *et al.*, 2011). Clean reads fully matching other RNAs, including repeat RNA, rRNA, snRNA, snoRNA, and tRNA, were excluded by blastn-short alignment (blast2.2.26+, ftp://ftp.ncbi.nih.gov/blast/executables/blast+/2.2.26/) against the Sanger RNA family database (Rfam 10.1, ftp://ftp.sanger.ac.uk/pub/databases/Rfam) (Gardner *et al.*, 2011). The remaining sequences were further aligned against miRBase (Release 20, http://www.mirbase.org/) (Kozomara and Griffiths-Jones, 2011) to discriminate conserved reads and non-conserved reads. A conserved read is defined as having no more than three mismatches with known miRNA sequences; otherwise reads are defined as non-conserved reads. As some well-known miRNAs cannot be identified to have miRNA* in a small RNA sequencing data set in some model plant species (Li *et al.*, 2011; Xie *et al.*, 2012), only novel miRNAs and their miRNA*s were required to co-exist in at least one sequencing library.

The newest expressed sequence tag (EST; 300 288) and Genome Survey Sequences (GSS; 62 820) databases were assembled with CAP3-based TGICL (ftp://ftp.tigr.org/pub/software/tgi/tgicl/). Given that only the draft D genome of *G. ramondii* in *Gossypium* sp. is available (Lin *et al.*, 2010; Paterson *et al.*, 2012; Wang *et al.*, 2012), the assembled databases of EST and GSS and the *G*. *ramondii* genome were used as the data source to identify miRNA precursors. The miRDeepFinder tool (http://www.leonxie.com/deepfinder.php) was used to identify miRNAs and their targets with the default parameter settings in the software (Xie *et al.*, 2012). Identification of miRNA precursor candidates was performed by all-to-all alignment to remove repeats. miRNA targets were predicted from the cotton annotated mRNA database at the NCBI and the assembled protein-coding EST contigs from the authors’s cotton EST database (www.leonxie.com) (Xie *et al.*, 2011).

Three released cotton degradome sequencing data sets (GSM1008997, seedlings; GSM1008999, hypocotyl; and GSM1061853, anthers) of upland cotton from the NCBI were used to validate the predicted miRNA targets using CleveLand4 with default parameters (Brousse *et al.*, 2014). As degradome sequencing is for the detection of the splice site on miRNA targets that generally occurs on the 10th and 11th nucleotides of mature miRNAs, no mismatches are allowed on these two nucleotides in degradome analysis (Schwab *et al.*, 2005). Finally, miRNA targets with a *P*-value of ≤0.05 were retained.

### Comparison of miRNA expression profiles in control, salt-, and drought-treated cotton seedlings

The abundance of all miRNAs was standardized to the transcript expression level per million reads (RPM). If the original miRNA expression in a library was zero, the normalized expression was adjusted to 0.01 according to a previous report (Murakami *et al.*, 2006). miRNA expression fold change in any two libraries was calculated with the formula, fold change=log_2_(treatment 1/treatment 2) (Marsit *et al.*, 2006). Pearson’s χ^2^ test was performed for determination of the significance of miRNA expression from two samples. The fold change and *P*-value were combined to determine the significance of the final miRNA expression. The expression difference level was defined as follows: extremely significant (**) if (fold change ≥1 or fold change ≤ –1) and *P*-value ≤0.01; significant (*) if (fold change ≥1 or fold change ≤ –1) and 0.05 ≥ *P*-value >0.01; and otherwise non-significant.

### Validation of miRNA expression profiles in control, salt-, and drought-treated cotton seedlings

Using the stem–loop quantitative reverse transcription–PCR (RT–PCR) method for assay of miRNA expression (Chen *et al.*, 2005), 13 miRNAs, comprising 10 conserved miRNAs (ghr-miR156a, ghr-miR157a, ghr-miR160a, ghr-miR166a, ghr-miR167b, ghr-miR171a, ghr-miR2911, ghr-miR394a, ghr-miR3954a, and ghr-miR395a) and three novel species-specific miRNAs (ghr-n65, ghr-n68, and ghr-n8) were randomly selected to validate miRNA expression of deep sequencing. Ten-day-old cotton seedlings in three biological replicates for qRT–PCR were treated in the same way as those for deep sequencing. qRT–PCRs were performed with an Applied Biosystems ViiA 7 Real Time PCR System for each reaction with three technical replicates. Pearson’s correlation test was performed to test the correlation significance of miRNA expression in the drought and salinity treatments relative to the control between qRT–PCR and deep sequencing with a *P*-value of 0.05.

### CitationRank-based literature mining

A large number of studies have been performed on salinity and drought stress in plant, in which many genes have been proposed or validated to be crucial for response to salinity and drought stress. To determine how the identified miRNAs are correlated with these genes, CitationRank-based text mining (Yang *et al.*, 2009) was first used to determine their importance to salinity and drought stress, respectively. In this step, briefly, the key words, ‘Salt AND Plant AND stress’ and ‘salinity AND Plant AND stress’, were used to retrieve salinity-related PubMed entries. Similarly, drought-related PubMed entries were obtained through the key words, ‘Drought AND Plant AND stress’ and ‘Water deficit AND Plant AND stress’. Retrieved PubMed entries that are from non-plant species were excluded according to the annotation in the Gene-to-PubMed data set, gene2PubMed, which was downloaded from the NCBI (ftp://ftp.ncbi.nlm.nih.gov/gene/DATA/). Considering that some genes in PubMed entries are homologous and also might be from different species, these genes were categorized as one functional gene. To this end, Orthomcl (Version 2.0, http://orthomcl.org/common/downloads/) (Li *et al.*, 2003) was used to search for orthologues among the genes in all retrieved PubMed entries with a threshold of 1e-20. It was hypothesized that a group of homologous genes should carry out a similar function. The CitationRank algorithm was followed to build up a co-existence matrix of genes and to compute the CitationRank value with an iteration of 1000. The whole process of CitationRank calculation was incorporated into a software package named RNAKER, which is freely available at http://leonxie.com/citationRank.php. Cytoscape was used to visualize the regulation network between cotton miRNAs and the cluster of coding homologues.

## Results

### High-throughput sequencing of control, salt-, and drought-treated small RNA libraries

**Fig. 1.**
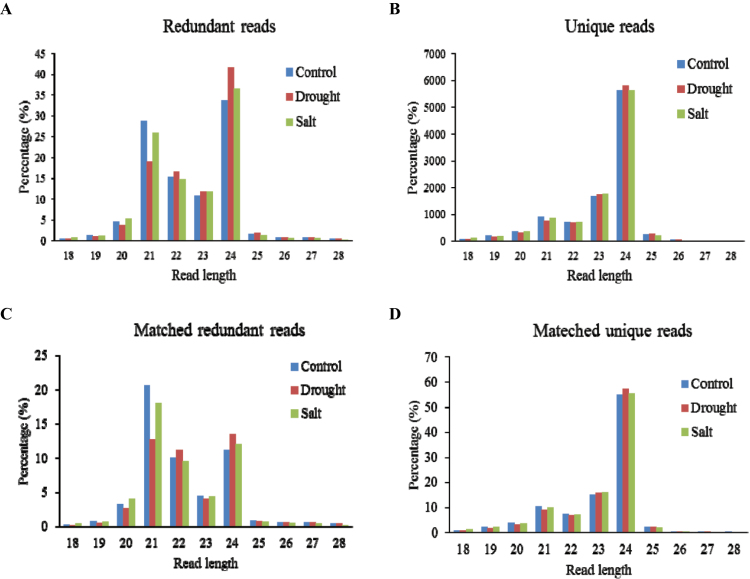
Size distribution of redundant and unique small RNA reads in cotton. (A and C) Size distribution of redundant small RNA reads from control, drought, and salt libraries. (B and D) Size distribution of unique small RNA reads from control, drought, and salt libraries. (C and D) Small RNA reads were fully mapped back to EST and GSS of upland cotton and the *G. ramondii* genome.

A total of 51 857 063 reads were generated from the three cotton small RNA libraries generated from salinity (18 808 997) and drought (16 938 676) treatment as well the control (16 109 390), representing a total of 16 126 755 unique sequences (Table 1). As the upland cotton genome is still not available, these sequence reads were first aligned against the GSS and EST databases of upland cotton. An average of 15.99% reads and 51.63% reads were fully (100%) matched back to the upland cotton data sets (EST and GSS) and the D genome of *G*. *ramondii*, respectively, resulting in a mean of 54.59% successful matches in upland cotton and *G. ramondii* (Table 1). Overall, the reads generated and the matched reads are similar in the three libraries. The Jaccard index was calculated for the 5000 most abundant small RNA reads in each library in order to evaluate the overall sequence similarity among the three libraries (Mohorianu *et al.*, 2011). The similarity between salt- and drought-treated libraries was 97.39% (Table 2). Furthermore, the two libraries showed a similar sequence similarity with the control library (control versus drought, 42.27%; and control versus salt, 46.94%), respectively. This indicates that some common small RNAs relatively rich in abundance might be readily induced to cope with abiotic stress in cotton, such as drought and salinity stress. All three libraries displayed similar distributions to other RNA families including rRNA (~1.34% for the unique and ~6.57% for the redundant reads), snRNA (~0.02% for the unique and ~0.01% for the redundant reads), snoRNA (~0.01% for the unique and ~0.00% for the redundant reads), and tRNA (~0.13% for the unique and ~1. 03% for the redundant reads) (Table 1). A similar size distribution for redundant reads, unique reads, and matched unique reads was observed in the three libraries, in which the 24 nucleotide reads account for the majority (Fig. 1). However, the matched redundant reads have the most reads in the 21 nucleotide class following by 24 nucleotides. The small RNA abundance and size in cotton were largely consistent with the results reported in *Arabidopsis* (Rajagopalan *et al.*, 2006) and rice (Wei *et al.*, 2011).

**Table 1.** Small RNA categorization in cotton^a^

	Unique (C)	Redundant (C)	Unique (D)	Redundant (D)	Unique (S)	Redundant (S)
Matched (E/G)	246 723 (4.31%)	2 740 263 (17.01%)	279 212 (3.65%)	2 714 775 (14.43%)	248 892 (3.90%)	2 800 217 (16.53%)
Matched (G)	1 775 856 (31.02%)	8 740 467 (54.26%)	2 314 399 (30.25%)	9 198 501 (48.90%)	1 965 288 (30.82%)	8 764 559 (51.74%)
Matched (A)	1 847 787 (32.27%)	9 229 827 (57.29%)	2 398 328 (31.35%)	9 719 543 (51.67%)	2 040 086 (31.99%)	9 281 862 (54.80%)
miRNA	29 145 (0.51%)	4 265 679 (26.48%)	31 229 (0.41%)	3 761 665 (20.00%)	31 343 (0.49%)	4 114 428 (24.29%)
rRNA	87 185 (1.52%)	1 230 970 (7.64%)	95 647 (1.25%)	1 117 486 (5.94%)	80 630 (1.26%)	1 038 386 (6.13%)
snRNA	1250 (0.02%)	2041 (0.01%)	1532 (0.02%)	2619 (0.01%)	1353 (0.02%)	2 338 (0.01%)
snoRNA	486 (0.01%)	749 (0.00%)	611 (0.01%)	1 148 (0.01%)	530 (0.01%)	746 (0.00%)
tRNA	8586 (0.15%)	182 011 (1.13%)	10 198 (0.13%)	201 042 (1.07%)	7410 (0.12%)	151 590 (0.89%)
Unan	5 598 655 (97.79%)	10 427 939 (64.73%)	7 511 572 (98.18%)	13 725 037 (72.97%)	6 256 412 (98.10%)	11 631 188 (68.67%)
Total	5 725 308	16 109 390	7 650 789	18 808 997	6 377 678	16 938 676

^*a*^ The number represented the raw data generated directly from deep sequencing;

C, control; S, salt; D, drought; matched (E/G), matched to EST and GSS of upland cotton; matched (G), matched to the genome of *G. ramondii*; matched (A), matched to EST and GSS of upland cotton and to the genome of *G. ramondii*; unan, unannotated.

**Table 2.** The similarity of the three cotton small RNA libraries: control, drought, and salt treatment

Jaccard index	Control	Drought	Salt
Control	–	42.27%	46.94%
Drought	42.27%	–	97.39%
Salt	46.94%	97.39%	–

### Identification of conserved miRNA families in cotton

**Fig. 2.**
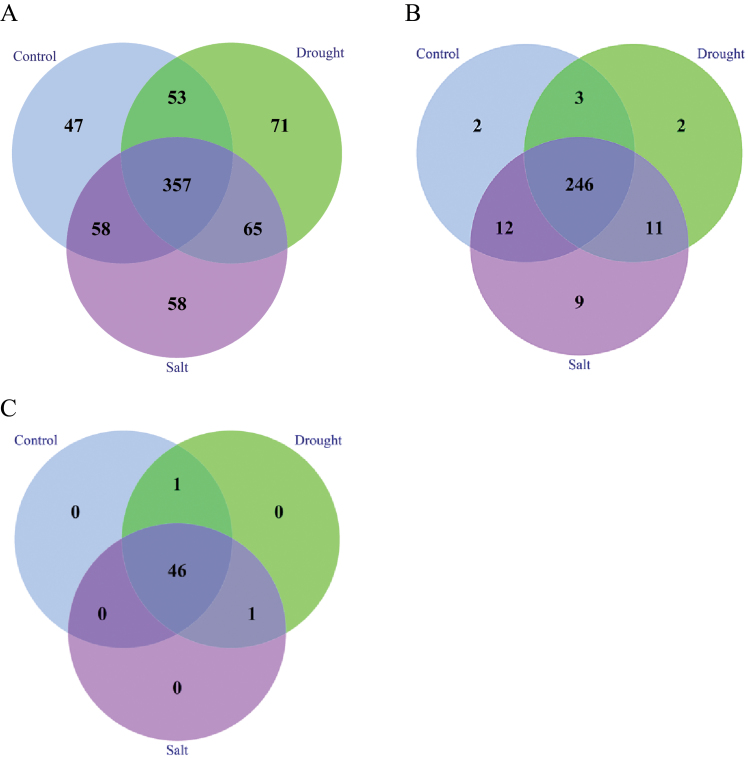
Distribution of miRNAs in control, drought, and salinity treatment. (A) Conserved miRNA families; (B) known miRNAs; (C) novel miRNAs.

miRNAs are well known to be highly conserved across species. According to alignment of all clean reads from three libraries against all known plant miRNAs in miRBase (Release 20) (Kozomara and Griffiths-Jones, 2011) with no more than three mismatches, a total of 709 known plant miRNA families were identified in cotton; out of these, 515, 546, and 538 families are from control, drought-treated, and salted-treated samples, respectively (Supplementary Table S1 available at *JXB* online). These miRNA families accounted for ~0.47% of the total unique read sequences and 23.59% of the total redundant read sequences on average (Table 1). Among these miRNA families, 71 and 58 miRNA families were specific to drought and salt treatment, respectively, whereas 47 miRNA families were only found in the control treatment (Fig. 2A). For example, miR1868 and miR2099 were expressed only in drought- and salt-treated samples, respectively (Supplementary Table S1). In addition, drought- and salt-treated libraries shared 65 miRNA families that did not occur in the control library. A total of 357 out of 709 miRNA families were identified in the three libraries, suggesting their key roles in maintaining normal biological activities, such as miR156/157, miR159, miR168, and miR172 (Table 3; Supplementary Table S1). Interestingly, all three libraries share similar most frequent miRNA families, including miR156, miR157, miR166, miR167, and miR3954. Pearson’s χ^2^ test showed that 565 out of 709 (79.69%) miRNA families were expressed differentially in the three libraries (*P*-value ≤0.05). Both fold change and the *P*-value were used to define the significance of expression (significant *, absolute fold change ≥1 and *P*-value ≤0.05; extremely significant **, absolute fold change ≥1 and *P*-value ≤0.01). It turned out that a total of 443 (62.48%) miRNA families showed a significant difference in expression in the pairwise comparison of the three treatments, including miR157, miR159, miR2948, and miR3694 (Table 3; Suppelementary Table S1). miR1854 and miR1148 had the largest fold change in both control versus drought and salt versus drought, up to ≥10 fold, indicating that drought stress strongly inhibited their expression. Similarly, miR1097 and miR5170 were expressed 5- to 7-fold less in the salt-treated sample than in the control and drought treatment.

**Table 3.** The expression of conserved miRNA families among control (C), drought (D), and salt (S) treatments

miRNA	miRNA normalization^*a*^			Fold change			Statistical significance			Expression significance		
	C	D	S	D/C	S/C	S/D	D versus C	S versus C	S versus D	D versus C	S versus C	S versus D
miR156	30 118.5	25 891.5	42 784.9	–0.44	0.43	0.88	**	**	**			
miR157	129 929.3	64 844.7	10 3178.0	–1.23	–0.41	0.82	**	**	**	**		
miR158	4.7	5.4	3.8	–0.04	–0.39	–0.36			*			
miR159	149.7	151.6	484.3	–0.20	1.62	1.83		**	**		**	**
miR160	130.9	132.2	65.5	–0.21	–1.07	–0.86		**	**		**	
miR161	4.7	5.2	3.8	–0.06	–0.35	–0.29						
miR162	73.9	53.2	71.4	–0.70	–0.12	0.58	**		**			
miR163	3.5	0.5	0.2	–3.11	–4.39	–1.28	**	**		**	**	
miR164	75.7	61.4	105.7	–0.53	0.41	0.94	**	**	**			
miR165	72.8	53.0	49.8	–0.68	–0.62	0.06	**	**				
miR166	13 680.8	9704.4	12 182.0	–0.72	–0.24	0.48	**	**	**			
miR167	3984.1	2999.4	3004.2	–0.63	–0.48	0.15	**	**				
miR168	1116.1	1017.5	1068.7	–0.36	–0.14	0.22	**	**	**			
miR169	176.7	128.6	101.8	–0.68	–0.87	–0.19	**	**	**			
miR170	0.6	1.3	0.8	1.03	0.39	–0.64	*			*		
miR171	166.1	179.4	129.1	–0.11	–0.44	–0.32	**	**	**			
miR172	477.9	394.1	316.5	–0.50	–0.67	–0.17	**	**	**			
miR173	2.3	0.0	0.0	–3.83	–3.83	0.00	**	**		**	**	
miR319	1.9	1.1	4.3	–1.08	1.09	2.17	*	**	**	*	**	**
miR390	237.7	230.3	215.1	–0.27	–0.22	0.05		**	**			
miR391	0.0	0.3	0.0	0.76	0.00	–0.76	*		*			
miR393	5.7	4.4	6.7	–0.61	0.16	0.78			**			
miR394	4.2	9.0	17.0	0.87	1.94	1.07	**	**	**		**	**
miR395	11.9	5.4	9.6	–1.35	–0.38	0.97	**	*	**	**		
miR396	387.2	274.8	363.8	–0.72	–0.16	0.56	**	**	**			
miR397	860.2	662.7	964.1	–0.60	0.09	0.69	**	**	**			
miR398	45.3	40.0	63.0	–0.40	0.40	0.81	*	**	**			
miR399	11.4	12.5	9.5	–0.09	–0.34	–0.25			**			
miR400	0.0	0.3	0.0	0.50	0.00	–0.50	*		*			
miR401	1.2	9.7	0.0	2.74	–2.95	–5.68	**	**	**	**	**	**
miR403	23.3	22.9	18.9	–0.25	–0.37	–0.13		**	**			
miR407	0.0	0.3	0.0	0.50	0.00	–0.50	*		*			
miR408	226.3	150.5	308.5	–0.81	0.37	1.19	**	**	**			**
miR413	0.0	0.0	0.6	0.00	1.80	1.80		**	**		**	**
miR414	8.4	3.1	9.6	–1.64	0.12	1.76	**		**	**		**
miR415	38.9	34.1	39.2	–0.41	–0.06	0.35	*		*			
miR416	3.9	0.1	0.0	–6.42	–4.60	1.82	**	**		**	**	
miR417	0.0	3.9	0.3	4.37	0.80	–3.57	**	*	**	**		**
miR418	17.6	20.8	21.7	0.01	0.23	0.21	*	**				
miR419	1.1	2.4	62.6	0.91	5.74	4.83	**	**	**		**	**
miR420	0.3	5.4	14.6	3.89	5.48	1.59	**	**	**	**	**	**
miR426	0.3	0.3	0.5	–0.45	0.53	0.98						
miR437	0.6	1.0	0.4	0.40	–0.66	–1.06						
miR440	0.0	0.0	0.6	0.00	1.80	1.80		**	**		**	**
miR442	2.2	0.0	1.1	–3.75	–1.03	2.73	**	*	**	**	*	**
miR443	5.4	5.3	5.1	–0.25	–0.14	0.10						
miR444	5.0	28.6	0.1	2.28	–5.48	–7.77	**	**	**	**	**	**
miR445	0.0	0.2	0.0	0.18	0.00	–0.18						
miR446	1.0	0.0	0.1	–2.62	–3.14	–0.52	**	**		**	**	
miR447	10.6	8.9	13.3	–0.48	0.25	0.73		*	**			
miR472	0.7	6.2	0.7	2.95	–0.02	–2.97	**		**	**		**
miR473	9.8	3.1	3.7	–1.87	–1.47	0.40	**	**		**	**	
miR474	5.1	7.0	8.3	0.24	0.64	0.40	*	**				
miR475	0.0	0.3	0.0	0.50	0.00	–0.50	*		*			
miR476	2.4	2.1	1.9	–0.41	–0.39	0.02						
miR477	37.9	24.2	32.8	–0.87	–0.28	0.59	**	*	**			
miR478	0.0	7.4	0.5	5.31	1.48	–3.83	**	**	**	**	**	**
miR479	0.3	0.2	0.3	–1.18	–0.14	1.04						
miR480	0.0	0.6	0.0	1.76	0.00	–1.76	**		**	**		**
miR482	37.5	27.2	29.8	–0.69	–0.40	0.28	**	**				
miR528	0.8	0.4	0.6	–1.15	–0.52	0.62				*		*
miR529	9.1	7.9	7.4	–0.42	–0.37	0.05						
miR535	1486.8	1097.5	1382.5	–0.66	–0.18	0.48	**	**	**			
miR774	415.3	365.6	421.2	–0.41	–0.05	0.36	**		**			
miR894	569.7	337.0	384.4	–0.98	–0.64	0.34	**	**	**			
miR2089	1578.8	1149.4	1585.8	–0.68	–0.07	0.62	**		**			
miR2911	621.8	762.6	477.7	0.07	–0.45	–0.52	**	**	**			
miR3476	640.4	562.9	535.0	–0.41	–0.33	0.08	**	**	**			
miR3954	69 714.5	82 391.4	64 928.4	0.02	–0.18	–0.19	**	**	**			
miR4377	2086.5	1551.5	2047.8	–0.65	–0.10	0.55	**	*	**			

^*a*^ All miRNA families with redundant abundance were normalized to transcript expression levels per million reads (RPM). If the original miRNA expression in a library was zero, the normalized expression was adjusted to 0.01 according to a previous report (Murakami *et al.*, 2006). miRNA expression fold change in any two libraries was calculated with the formula, Fold change=log_2_(treatment 1/treatment 2) (Marsit *et al.*, 2006). A 2×2 contingency table was used to perform Pearson’s χ^2^ test for significance of miRNA expression from two samples. Fold change and the *P*-value were combined to determine the final miRNA expression significance. The expression difference level was defined by the following rules: extremely significant (**) if (fold change ≥1 or fold change ≤ –1) and *P*-value ≤ 0.01; significant (*) if (fold change ≥1 or fold change ≤ –1) and 0.05 ≥ *P*-value > 0.01; otherwise non-significant.

### Identification of miRNA precursors and novel miRNAs

To avoid possible sequencing errors, only a total of 1 284 088 unique small RNAs with at least three reads in one of the three libraries were used to search for miRNA precursors. As the upland cotton genome is not available and it consists of an A and D genome, the search for miRNA precursors was performed on EST and GSS databases of upland cotton and the D genome of *G*. *ramondii*, respectively. On average, the search yielded 3 602 105 and 2 798 118 one hundred percent nucleotide match hits per library against the assembled EST/GSS databases and *G. ramondii* genome, respectively. Finally, after removing repeated precursors, a total of 337 miRNAs with precursors were obtained, comprising 289 known miRNAs and 48 novel miRNAs (Supplementary Table S2 at *JXB* online). Of these miRNAs, there are 31 from ESTs, six from EST contigs, 13 from GSS, and 10 from GSS contigs. A total of 277 out of 337 (82%) were identified from the *G. ramondii* genome. The 277 *G. ramondii* genome-derived miRNA precursors were aligned against the data sets of EST and GSS of upland cotton to see if these miRNAs could have a homologue sequences with at least 95% identity in upland cotton. It turned out that 21, nine, 29, and eight miRNA precursors are homologous to those from EST, EST contigs, GSS, and GSS contigs, respectively (Supplementary Table S2). Among the 337 identified miRNAs, there are at least 121 conserved miRNAs and four novel miRNAs that have been recently reported in upland cotton by Xue *et al.* (2013) and Li *et al.* (2012). Moreover, the mature miRNAs ghr-miR477*, ghr-miR2608*, ghr-miR827*, and ghr-miR166j* are the same as the mature miRNA of novel_mir_986, novel_mir_1398, novel_mir_50, and novel_mir_848, respectively, of Xue *et al.* (2013). For all 48 newly identified novel miRNAs, no homologues was found in other plant species, indicating that these novel miRNAs might be cotton specific.

The 337 miRNAs consisted of 154 miRNA families, including 84 known miRNA families and 44 novel miRNA families. A total of 106 (30.99%) miRNAs have only one member, whereas the other miRNA families have 2–16 members. The largest miRNA family is miR5528, with up to 16 members, followed by miR166 (13 members), miR169 (13 members), miR171 (11 members), and miR172 (10 members). Interestingly, there are five members for the newly identified miRNA ghr-n36 family.

A total of eight miRNA clusters were also found in the identified miRNAs, of which two and six clusters were from the EST/GSS of upland cotton and *G. ramondii* genome, respectively (Supplementary Table S3 at *JXB* onlime). Normally, miRNA*s are thought to degrade shortly after miRNA maturation and are present at an extremely low level (Ambros, 2004). Recently, miRNA*s were also found to be bona fide miRNAs, since they also participate in negative gene regulation with the same mechanism as miRNAs (Kozomara and Griffiths-Jones, 2011). Here, 21 miRNA*s that were generally expressed more than the corresponding mature miRNAs were also identified in at least one sequenced library, such as ghr-miR156*, ghr-miR7495*, ghr-miR166j*, and ghr-miR169c*. In addition, some miRNA*s have similar expression abundance to their mature miRNAs; it is thought that these miRNAs are likely to be functional in cotton species or in response to drought and salinity stress.

### miRNA expression in response to drought and salt treatment

**Fig. 3.**
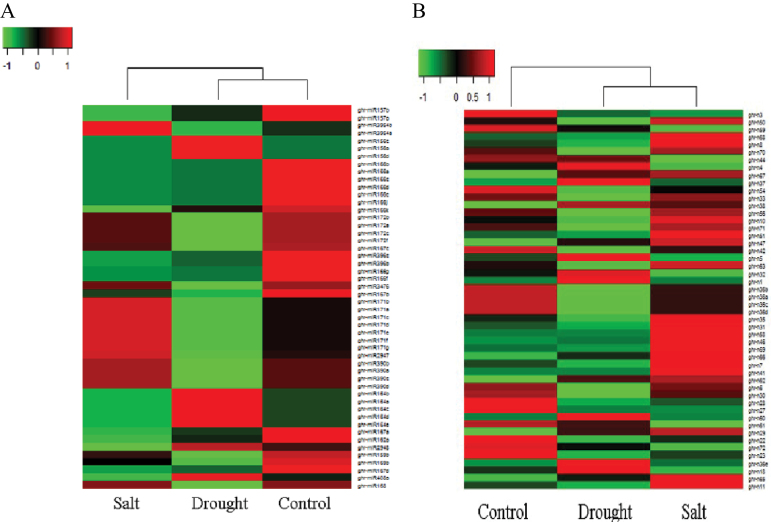
Heatmaps of (A) the top 50 most abundant conserved miRNAs and (B) the top 50 most abundant novel miRNAs in control, salt, and drought libraries in cotton. Red, up-regulated; green, down-regulated.

The overall expression of identified miRNAs was similar between control and salinity tratment, but not between control and drought treatment (data not shown). According to the heatmap for the 50 most abundant known miRNAs and novel miRNAs that represent 99.80% and 99.33% of the total expression abundance of known miRNAs and novel miRNAs, respectively, control treatment is closer to drought treatment for known miRNAs (Fig. 3A), whereas overall novel miRNAs in drought treatment were expressed more similarly to salt treatment relative to control treatment (Fig. 3B).

A total of 292 out of 337 (86.6%) miRNAs were expressed in all of the three treatments, comprising 246 known miRNAs and 46 novel miRNAs (Fig. 2B). Two and nine miRNAs were found to be expressed specifically in drought and salt treatments, respectively. Eleven known miRNAs and one novel miRNA co-exist in both drought and salt treatments, but not in control treatment. Similarly, 15 known miRNAs and one novel miRNA were specific to only control and drought treatments, whereas nine known miRNAs and one novel miRNA were specific only to control and salt treatments (Fig. 2B, C). The expression specificity of these miRNAs in the three treatments indicates differential roles in response to salt and drought stress.

According to Pearson’s χ^2^ test, 155 (50.0%) miRNAs were expressed differentially amongst control, drought, and salt treatments (*P*-value ≤0.05), including 140 known miRNAs and 15 novel miRNAs, such as ghr-miR156a, ghr-miR166a, ghr-miR168, and ghr-n3 (Supplementary Table S2 at *JXB* online). Based on the fold change (≥1) and *P*-value (≤0.05), a total of 77 miRNAs were found to have a significant difference in expression in any two samples, including ghr-miR160b, ghr-miR399c, ghr-miR172i, ghr-n3, and ghr-n50.

Besides drought/salinity-specific miRNAs, some miRNAs were significantly up-regulated or down-regulated by drought or salt treatment (Supplementary Table S2 at *JXB* online). For instance, ghr-miR157a/b, ghr-miR166a-j, ghr-miR167a, ghr-miR172a/b/c/f, and ghr-miR396g were down-regulated in both drought and salinity treatments when compared with control treatment, whereas ghr-miR394a-d, ghr-miR160b/c, ghr-miR393c, and ghr-miR5340 were up-regulated in drought and salinity treatments. Likewise, some miRNAs were up-regulated or down-regulated under drought or salt stress and, vice versa, down-regulated or up-regulated in the other non-control treatment, such as ghr-miR156a/c/d, ghr-miR408a, ghr-miR2911, and ghr-miR3954a/b.

### Validation of miRNA expression by qRT–PCR

**Fig. 4.**
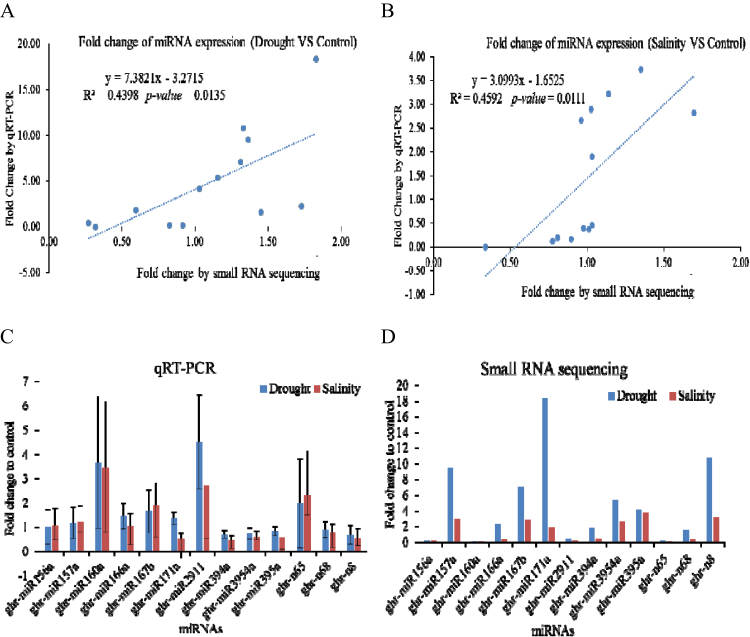
Validation and comparison of the expression of 13 cotton miRNAs between qRT–PCR and small RNA sequencing. miRNA expression correlation between qRT–PCR and small RNA sequencing (A, drought versus control; B, salinity versus control). Fold change of miRNA expression relative to the control sample (C, qRT–PCR; and D, small RNA sequencing). qRT–PCR assay of the expression of each miRNA was performed on 10-day-old cotton seedlings in three biological replicates: control, drought, and salinity. The bar represents the standard deviation.

To test the reliability of deep sequencing, 13 miRNAs (10 conserved miRNAs and three novel miRNAs) were randomly selected for stem–loop qRT–PCR. Compared with the control miRNA expression, the expression of most of the 13 miRNAs in the drought and salinity treatments had a similar tendency between deep sequencing and qRT–PCR, respectively (Fig. 4). Pearson’s correlation test showed that expression of the 13 miRNAs relative to the control exhibited significantly positive correlations between deep sequencing and qRT–PCR (*R* ^2^=0.4398 and *P*-value=0.0135; and *R* ^2^=0.4592 and *P*-value=0.0111) (Fig. 4A, B). Therefore, qRT–PCR-based validation indicated that the deep sequencing used is reliable in quantifying miRNA expression abundance in cotton. However, the *R* ^2^ was a little low due to the fold change being slightly different among different tested miRNAs, possibly due to, at least partially, the sensitivity of the deep sequencing technology. Overall, qRT–PCR showed higher fold change of miRNA expression in drought versus control and salinity versus control than the changes in small RNA sequencing (Fig. 4C, D). It was inferred that this might be due to exponential amplification in PCR that might elevate real miRNA expression. In all the expression of 13 randomly selected miRNAs was validated by qRT–PCR, except miR394a in drought treatment, which, based on deep sequencing, was up-regulated, but based on qRT–PCR was down-regulated. One of the potential reasons may be because the samples came from different treatment times which may cause a slight difference.

### miRNA target identification and validation

To predict cotton miRNAs targets, two upland cotton mRNA data sets were utilized, cotton mRNA databases (2493 sequences) in the NCBI and the annotated cotton EST database (20 307 coding sequences) (Xie *et al.*, 2011). After strict filtration with a series of miRNA target features, a total of 1895 unique coding genes were predicted to be targets of 271 conserved miRNAs and 20 novel miRNAs, consisting of a total of 5430 miRNA–target pairs. Of these targets, 748 and 1147 are from the mRNA database and assembled EST database, respectively (Supplementary Table S4 at *JXB* online). Degradome sequencing, also known as PARE (parallel analysis of RNA ends), is used extensively to discover *in vivo* miRNA targets through detecting cleaved miRNA targets from degradome data (Addo-Quaye *et al.*, 2009). Based on three sets of cotton degradome sequencing data from seedlings, hypocotyl, and anthers, 114 miRNA–target pairs were further verified in the miRNA prediction result, which include 60 miRNAs and 55 coding genes (Suppementary Table S4). Many of these degradome-validated miRNA–target pairs are well known in other species, such as ghr-miR156–SBP (squamosa promoter-binding protein), ghr-miR160–auxin response factor, ghr-miR168–argonaute protein, and ghr-miR172–AP2 (Backman *et al.*, 2008).

miRNA target prediction revealed at least 1019 important genes, which are involved in a variety of biological processes, including fibre development and stress response. These genes were manually classified into 11 major groups based on previous reports, including apoptosis, cell cycle, fibre development, gossypol biosynthesis, stress response, signal transduction, and transcription factors (Table 4; Supplementary Table S4 at *JXB* online). A majority of miRNAs were predicted to target genes that are associated with stress response, transcription factors, metabolism, and fibre development (Table 4). For example, based on the target prediction, 151 miRNAs might regulate 229 stress response-related genes, whereas 217 transcription factors might be targets of 183 miRNAs.

**Table 4.** Stress-, resistance-, and fibre-related miRNAs, miRNA targets, GO terms, and KEGG pathways in cotton

Function type	miRNAs	Targets	Cellular component	Biological process	Molecular function	Pathway
Apoptosis	14	4	3	4	3	
Cell cycle	13	7	1	7	3	
Cell migration	23	15	2	2	3	
Circadian clock	2	3	1	12	4	1
Development	46	17	11	32	20	11
Fibre development	163	210	41	132	94	21
Gossypol biosynthesis	3	1	1	1	1	
Metabolism	181	239	48	198	187	57
Signal transduction	110	104	27	85	46	6
Stress response	151	229	40	136	104	22
Transcription factor	183	217	29	131	47	4

Generally, miRNAs are well known to be highly conserved across different species, even for miRNA regulatory function. In the target prediction result, some established miRNA targets were also similarly identified in cotton. For example, ghr-miR156b/c/d was predicted to target SBP transcription factors (contig7220, contig7221, and contig20213), which have been validated to be miR156 targets and are involved in flower development in *Arabidopsis* (Brousse *et al.*, 2014) and rice (Schwab *et al.*, 2005) (Supplementary Table S4 at *JXB* online). Through negatively regulating expression of auxin response factor ARF-10, -16, and -17 in *Arabidopsis*, miR160 is important for various plant development processes including seedlings, embryo development, and inflorescences (Xue *et al.*, 2013). It was also found and validated that ghr-miR160b/c might target ARFs in cotton, indicating that ghr-miR160b/c might participate in cotton seedling development and seedling resistance to salinity and drought stress. Additionally, some other traditional conserved miRNA–target pairs were also identified in cotton, such as ghr-miR168–AGO1, ghr-miR164a–NAC, and ghr-miR172c–AP2 (Supplementary Table S4).

Abiotic stress including drought and salinity stress always induces metabolic rearrangements and regulatory networks in terms of osmotic stress, disorganized membrane, low activity or denatured protein, and accumulation of excessive reactive oxygen species (ROS) (Krasensky and Jonak, 2012). Of these, ROS overproduction in plants is highly reactive and toxic, causing damage to protein, lipids, carbohydrates, and DNA, and finally resulting in oxidative stress. Fortunately, plants possess various enzymes against oxidative stress, including SOD, catalase (CAT), ascorbate peroxidase (APX), glutathione reductase (GR), monodehydroascorbate reductase (MDHAR), dehydroascorbate reductase (DHAR), glutathione peroxidase (GPX), and guaicol peroxidase (GOPX) (Li *et al.*, 2012). There are many ROS-related genes which have identified to be miRNA targets in cotton. Cu/Zn SOD (FJ415203.1) might be targeted by ghr-miR398b. In fact, miR398 has a conserved function that was validated to act on two closely related Cu/Zn SODs (cytosolic CSD1 and chloroplastic CSD2) and detoxify intracellular superoxide radicals in *Arabidopsis* (Sunkar *et al.*, 2006). This suggests that ghr-miR398b might be involved in response to stress in cotton seedlings due to salinity and drought through modulating its target, Cu/Zn SOD. Similarly, cytosolic APX (EU244476.1, FJ793812.1, and EF432582.1) might be targeted by ghr-miR447a and ghr-miR6190 in cotton (Supplementary Table S4 at *JXB* online). Furthermore, some specific drought/salt-responsive proteins which might be miRNA target candidates in cotton were also found, such as salt overly sensitive protein 2a (ghr-miR6190) (Addo-Quaye *et al.*, 2009).

**Fig. 5.**
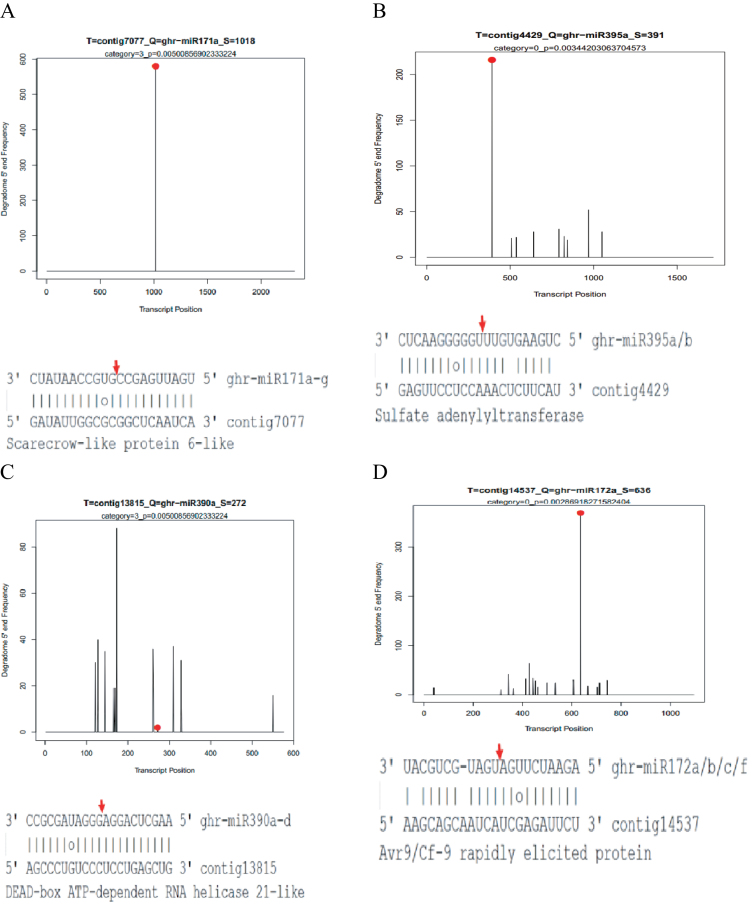
Cotton miRNA target alignment and its T-plot validated by degradome sequencing. (A) ghr-miR171a-g and contig7077 (Scarecrow-like protein 6-like); (B) ghr-miR390a-d and contig13815 (DEAD-box ATP-dependent RNA helicase 21-like); (C) ghr-miR395a/b and contig4429 (sulphate adenylyltransferase); and (D) ghr-miR172a/b/c/f and contig14537 (Avr9/Cf-9 rapidly elicited protein). Both the arrow and the dot represent the splice site on the miRNA target.

Based on the degradome sequencing data, it was possible to confirm some stress-related miRNA targets, including ghr-miR171a-g and contig7077 (scarecrow-like protein 6-like) (Fig. 5A), ghr-miR395a/b and contig4429 (sulphate adenylyltransferase) (Fig. 5B), ghr-miR390a-d and contig13815 (DEAD-box ATP-dependent RNA helicase 21-like) (Fig. 5C), and ghr-miR172a/b/c/f and contig14537 (Avr9/Cf-9 rapidly elicited protein) (Fig. 5D). For instance, low expression of osmotically responsive gene 4 (LOS4), a DEAD box RNA helicase gene, was found to be essential for mRNA export and important for development and stress responses in *Arabidopsis*; mutation of this gene could enhance cold stress induction of the master regulator of cold tolerance, C-repeat binding factor 2 (CBF2), and its downstream target genes (Gong *et al.*, 2005). Sulphur is a macronutrient that is necessary for plant growth and development, involved in assimilation of cysteine, methionine, glutathione, and other sulphur-containing metabolites (Liang *et al.*, 2010). It has been reported that sulphur metabolism plays significant roles in drought stress signalling transduction, since primary and secondary sulphur metabolism should be co-ordinated until a certain complex balance is achieved in plants (Chan *et al.*, 2013). To date, miRNA395 has been validated to participate in sulphur metabolism by targeting ATP-sulphurylase (Liang *et al.*, 2010) and sulphate transporter (Allen *et al.*, 2005). Both the miRNA target prediction and degradome sequencing analysis showed that ghr-miR395a/b is also likely to be involved in sulphur metabolism by regulating sulphate adenylyltransferase in cotton. Thus, these stress-related miRNAs and their targets might also play roles in response to drought and salinity stresses.

miRNAs not only target genes associated with drought and salinity stress but also genes involved in cotton fibre development. The fibre-related function involves fibre cell initiation, fibre-related carbohydrate metabolism, cellulose biosynthesis, fibre cell elongation, and some other important transcription factors (Supplementary Table S4 at *JXB* online). A total of 163 miRNAs were predicted to take part in fibre development by targeting 210 unique genes in cotton (Table 4; Supplementary Table S4). For instance, at least 15 cellulose synthase or cellulose synthase-like proteins might be targets of 34 miRNAs including ghr-miR166m, ghr-miR167b, ghr-miR169k, and ghr-miR172j. A MYB transcription factor, CAPRICE (*CPC*), was initially identified to be a negative regulator of non-root hair cells and later also found to play a role in inhibiting leaf trichome development in *Arabidopsis* (Backman *et al.*, 2008). Here, it was found that ghr-miR447a and ghr-miR5255a/b/c/e/f/g/h might target *CPC*, indicating that ghr-miR447a and ghr-miR5255a/b/c/e/f/g/h may play a role in root development to respond to drought and salinity stress or fibre development by regulating *CPC* in cotton (Supplementary Table S4).

### GO and KEGG pathway analysis

GO-based analysis allows the determination of which GO terms (biological process, molecular function, and cellular component) a gene belongs to (Ashburner *et al.*, 2000). Therefore, GO-based analysis could provide more ideas on understanding miRNA function. A total of 274 miRNAs (256 conserved miRNA and 18 novel miRNAs) and their 1252 targets were classified into 557 molecular functions, 729 biological processes, and 188 cellular components (Supplementary Table S5 at *JXB* online). At least 151 miRNAs and their 229 targets that are associated with stress response were able to be categorized into 104 molecular functions, 136 biological processes, and 40 cellular components (Table 4). Fifty-four miRNA–target pairs belong to the biological process of response to salt stress (GO:0009651), involving ghr-miR156e, ghr-miR162b, ghr-miR169h, ghr-miR172e, ghr-miR396h, and ghr-miR399i. Similarly, at least 35 miRNA–target pairs belong to the biological process of response to desiccation (GO:0009269) and response to water deprivation (GO:0009414), such as ghr-miR159b, ghr-miR166h, ghr-miR399i, ghr-n26, and ghr-miR399f. Many of the classified biological processes were associated with signal transduction, such as auxin metabolism (GO:0009850) and biosynthesis (GO:0009851), ethylene-mediated signalling pathway (GO:0009873), response to biotic stimulus (GO:0009607), cytokinin metabolism (GO:0009690) and biosynthesis (GO:0009691), and jasmonic acid metabolism (GO:0009694) and biosynthesis (GO:0009695) (Supplementary Table S5).

KEGG-based analysis allows enrichment of 159 miRNAs and 235 targets to 93 pathways, including photosynthesis (ath00195), glycolysis/gluconeogenesis (ath00010), oxidative phosphorylation (ath00190), biosynthesis of plant hormones (ath01070), and starch and sucrose metabolism (ath00500) (Supplementary Table S6 at *JXB* online). Twenty-two out of 93 pathways might interact with the stress response through 151 miRNAs and their 229 targets. Interestingly, many of these miRNAs and targets are involved in the pathways that metabolize and biosynthesize some intermediate products important for fibre development, such as glucose, sucrose, starch, and fatty acid.

### Text mining drought/salinity-responsive genes

A large number of genes have been identified in response to drought and salinity stress, and details exist in a vast amount of literature. Currently, there is no database that summarizes genes associated with response to drought and salinity stress. In order to investigate how the identified miRNAs act on these reported stress-responsive genes, it was hypothesized that a gene frequently mentioned or cited in reraltion to a certain topic or in a certain context should be an important gene for that topic or context. Such genes were named frequent genes. In addition, under the same topic or context, a gene co-existing with a frequent gene in the literature should be more important than other genes that exist singly in one paper or co-exist with an infrequent gene. The idea was successfully applied to sort the importance of genes to serious adverse drug reaction (SADR), known as the CitationRank algorithm (Yang *et al.*, 2009). Based on this idea, the algorithm was first implemented with PERL and then it was applied to rank a gene’s relevance to drought and salt stress. After searching with keywords of drought and salinity and discarding the genes and PubMed entries from non-plant species (see the Materials and methods), a total of 595 and 1078 effective coding genes are retrieved from 228 and 419 effective PubMed entries under the context of salinity and drought, respectively (data not shown). These genes are from 12 (salinity) and 15 (drought) plant species, respectively. Considering that these coding genes are probably homologous in different species, Orthomcl was used to cluster them and then a CitationRank value was calculated for each cluster. The salinity context-based and drought context-based genes were clustered to 351 and 918 groups with a cut-off E-value of 1e-20, respectively. To build the connection between context-based genes and identified miRNAs and targets, only the protein sequences of the longest genes in each cluster were compiled together as subject data sets, and a BLASTX alignment with miRNA targets under the cut-off E-value of 1e-20 was then performed.

In the CitationRank result, the top five ranked genes in the drought context are MYB3R transcription factor, serine/threonine-protein kinase EDR1, SKP1-like protein 18, aquaporin PIP1-5, and ABA receptor PYL7, corresponding to 27 miRNAs and 14 targets, such as ghr-miR156e, ghr-miR172g, ghr-miR447a, ghr-miR1876a, ghr-n6, contig11235 (MYB73 transcription factor), and GU207868.1 (serine/threonine protein kinase 1) (Supplementary Table S7 at *JXB* online). The genes including Granulin repeat cysteine protease, alcohol dehydrogenase 1, glycine-rich RNA-binding protein 3, gibberellin 2-beta-dioxygenase 7, and allene oxide cyclase 3, were ranked the top five in the salinity context, involving 11 miRNAs and eight targets (Supplementary Table S7). Interestingly, five miRNAs (ghr-miR5284, ghr-miR6158a, ghr-miR6158b, ghr-miR6190, and ghr-miR6424e) were associated with top-ranked genes in both a drought and salt context, implicating that they are drought/salinity-responsive miRNAs.

## Discussion

### Differentially expressed miRNAs involved in abiotic stress response

Under drought and salt stress, many conserved and novel miRNAs were expressed differentially; some miRNAs were even specifically expressed in drought and/or salt treatment. The miR156/157 family are the most abundant miRNAs in all three treatments, accounting for 60% of total miRNA reads (Table 3; Supplementary Table S1 at *JXB* online). miR156 and miR157 were down-regulated in drought treatment by 0.44- and 1.23-fold, respectively, when compared with the control. However, miR156 was up-regulated (0.43-fold) and miR157 was down-regulated (0.41-fold) by salt treatment (Table 3; Supplemetnary Table S1). Both miR156 and miR157 in cotton root and leaf were reported to be down-regulated at a high concentration of salt (>2.5%) and with the increase of PEG (drought) in a dose-dependent manner (Wang *et al.*, 2013). The present result was also largely consistent with the results. However, miR156 was up-regulated in salt treatment relative to the control. This might be caused by the fact that miR156 expression in the present results represents a miR156 family, but not a particular miR156 member. miR156/157 is well known to target the SPL transcription factor negatively in plants, and miR156/157 overexpression results in a delayed onset of adult traits and flowering in *Arabidopsis* (Park *et al.*, 2010). Overproduction of miR156 (Corngrass1, *Cg1*) caused an extension of the juvenile vegetative phase in maize (Aharon *et al.*, 2003). Current research on miR156/157 mainly focuses on its role in morphology change and regulation of blooming. Here evidence was provided from small RNA sequencing that drought and salinity stress disturb miR156/157 expression, indicating a novel role for miR156/157 in response to drought and salinity stress.

NF-YA (GmNFYA3) of the NF-Y complex in soybeans inducible by ABA and abiotic stresses including drought, NaCl, and cold. Overexpression of GmNFYA3 in *Arabidopsis* leads to reduced leaf water loss and enhanced drought tolerance, and elevates its sensitivity to high salinity and exogenous ABA. An *in vivo* experiment in tobacco showed that miR169 directs GmNFYA3 mRNA cleavage (Xue *et al.*, 2009). In cotton, it was predicted that NF-YA3 (contig16841 and contig22907) is the target of ghr-miR169. Additionally, compared with the control, ghr-miR169a/b/c expression in drought and salinity treatment was significantly down-regulated by 0.04- to 0.93-fold. In contrast, ghr-miR169d/e/f/g were significantly up-regulated in drought and salinity treatment by 0.04- to 0.85-fold. Ghr-miR169i/j expression was inhibited in drought treatment and up-regulated in salinity treatment. Therefore, it is inferred that at least ghr-miR169a/b/c might play a positive role in combating drought and salinity stress by acting on NF-YA3 in cotton.

Plant aquaporin proteins are a class of the large major intrinsic protein family, which are well known to play a role in transport through biological membranes of diverse small molecules including water and other small nutrients (Park *et al.*, 2010). In addition to a role in absorption and transportation of water and nutrients, aquaporin is also found to be involved in a series of abiotic stresses, such as drought and cold stress. For example, overexpression of a plasma membrane aquaporin in transgenic tobacco improves plant vigour under favourable growth conditions but not under drought or salt stress, since symplastic water transport via plasma membrane aquaporins has a deleterious effect during drought or salt stress (Aharon *et al.*, 2003). Recently, Rh-TIP1, a TIP-type aquaporin gene isolated from rose, was found to be repressed by treatment with both ethylene and water deficit (Xue *et al.*, 2009). Here it was identified that 20 miRNAs might target 22 aquaporin proteins in cotton, of which two miRNA–target pairs (ghr-miR4371 and contig780; and ghr-miR4371 and BK007054.1) were also detected by degradome sequencing analysis (Supplementary Table S4 at *JXB* online). Thus, aquaporins might be involved in response to drought and salinity stress in cotton by being an miRNA target, such as of ghr-miR4371.

Transgenic rice plants overexpressing miR393 are more sensitive to salt and alkali treatment, suggesting that miR393 is a negative regulator in response to salt and alkali stress by targeting abiotic-related genes (Gao *et al.*, 2011). In this study, an opposite result was obtained, in that ghr-miR393a/b/c/d/e expression was up-regulated with drought and salinity treatment. However, the predicted ghr-miR393 targets in cotton are also stress-related genes, as well several hormone-responsive genes, including NADPH:cytochrome P450 reductase (CPR1), class III peroxidase (POX4), protein AUXIN SIGNALING F-BOX 3, and TIR1 (TRANSPORT INHIBITOR RESPONSE 1). Therefore, if miR393 in cotton is also an negative stress-combating regulator as it is in rice, one possible explanation is that the up-regulation of miR393s in cotton seedlings in drought and salinity stress contributes to enlarging the stress signal in cotton and then triggers a more efficient or powerful pathway to counter drought and salinity stress by signal transduction cross-talk, probably by an auxin-related pathway.

### miRNAs and targets important for fibre development

Cotton fibre development and maturation determine fibre yields and quality. Therefore, particular attention was paid in this study to miRNAs and targets that are related to cotton fibre development. Interestingly, there are many identified miRNAs and their targets that are likely to play crucial roles in fibre development and maturation. MYB transcription factors have been known to play a role in promoting the differentiation of ovule epidermal cells into the elongated cotton fibre. Recently, GhMYB25-like, an R2M3 MYB, was newly identified as a key factor in early cotton fibre development, since GhMYB25-like silencing resulted in cotton plants with fibreless seeds (Walford *et al.*, 2011). Three GhMYB25a or GhMYB25-like (AF336283.1, AY464054.1, and HM134084.1) were detected to be targeted by ghr-miR4370, ghr-miR5565a/c, and ghr-miR6158a/b in cotton, indicating that the five miRNAs might be involved in early fibre development. In addition, of the large MYB family, there are many other MYB members that were also reported to be important during fibre development, such as MYB2 (Wang *et al.*, 2004) and MYB109 (Pu *et al.*, 2008). This suggests that MYB transcription factors are so important that their function in regulation should receive more atttention in understanding fibre development. A total of 40 miRNAs targeting at least 25 MYB transcription factors, such as ghr-miR156e, ghr-miR159a, ghr-miR162b, ghr-miR167b, ghr-miR169b, and ghr-miR172a, were uncovered. A recent study shows that two miRNAs (miR828 and miR858) target MYB2, which may play a role in fibre development (Guan *et al.*, 2014). In addition to the genes involved in fibre cell initiation and fibre early development, many identified miRNAs targeting a batch of genes that are related to fibre elongation and maturation were also found. For example, after fibre elongation, the fibre cell enters into secondary cell wall formation that is characterized by massive synthesis of cellulose comprising multiple β-1,4-glucan chains. Cellulose synthase is responsible for glucan chain elongation (Somerville *et al.*, 2004). Therefore, cellulose synthase is an indispensable part of fibre maturation. In the present study, at least 17 cellulose synthases were predicted to be the targets of 35 miRNAs in cotton, suggesting that these miRNAs and targets can be utilized to study their roles in fibre development. Overall, the result provided a good data resource for better understanding of fibre development. Several other studies also show that many miRNAs were differentially expressed during cotton fibre initiation and development (Pang *et al.*, 2009; Chen *et al.*, 2013; Gong *et al.*, 2013; Zhang *et al.*, 2013).

### Top-ranked genes and miRNAs for drought and salinity response

**Fig. 6.**
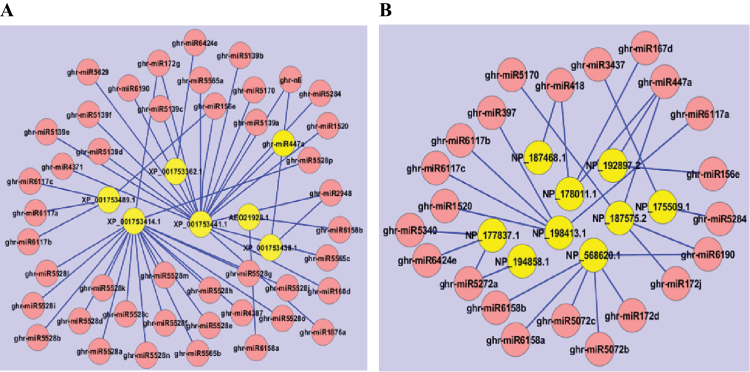
Top-ranked miRNA regulation networks involved in (A) drought response and (B) salinity response in cotton.

Using the CitationRank-based algorithm, coding genes relevant in the context of drought and salinity stress in plants were first sorted out from each other. MYB3R was ranked to be the most important gene in the drought context, being targeted by 10 miRNAs in cotton (Fig. 6A). Recently, increasing research has reported that MYB is associated with drought tolerance in plants. For instance, NbPHAN, an R2R3-type MYB gene in tobacco whose silencing led to severe wilting and an increased rate of water loss in tobacco leaf, was considered to play a positive role in addressing drought stress (Huang *et al.*, 2013). As described in the discussion above, MYB is also crucial for fibre development. Therefore, a related regulation mechanism between miRNA and MYB transcription factor in cotton is promising to serve a dual purpose, namely fibre development and drought tolerance. For the salinity response, genes including granulin repeat cysteine protease, alcohol dehydrogenase 1, glycine-rich RNA-binding protein 3, and gibberellin 2-beta-dioxygenase 7 were determined to be ranked high in terms of importance for salinity response (Fig. 6B). Interestingly, there are many reports showing that in fact granulin repeat cysteine protease did not associate with salinity response but rather with programmed cell death in plants (Yamada *et al.*, 2001; Chen *et al.*, 2006; Jiang *et al.*, 2007; Andeme Ondzighi *et al.*, 2008). Salinity stress might more readily trigger programmed cell death in plants through cysteine protease. However, the ranking result is based merely on literature text mining and more experimental evidence is needed in the future. Overall, the result of the ranking of relevance for the context of drought and salinity stress provides an idea of the gene/miRNA importance in drought and salinity stress in cotton, which could contribute to future cotton drought/salt tolerance research.

## Supplementary data

Supplementary data are available at *JXB* online.

Table S1. Summary of miRNA family comparison among control, salt, and drought libraries in cotton.

Table S2. Characteristics of all conserved miRNAs, including miRNA chromosome location, read number, and their precursor sequence.

Table S3. miRNA clusters in cotton and their expression among different treatments.

Table S4. miRNA targets for conserved and species-specific miRNAs in cotton.

Table S5. GO classification of identified miRNA families in cotton.

Table S6. Gene pathway analysis for cotton miRNA targets based on GO and KEGG analysis

Table S7. CitationRank results on gene clusters involving in drought and salinity treatment.

Supplementary Data
