# Supplementary material for: Deep sequencing reveals important roles of microRNAs in response to drought and salinity stress in cotton
Source: J Exp Bot. 2014 Nov 4;66(3):789–804. doi: 10.1093/jxb/eru437 (PMC4321542; doi:10.1093/jxb/eru437)
Supplement: Supplementary Data [file supp_66_3_789__index.html]

Deep sequencing reveals important roles of microRNAs in response to drought and salinity stress in cotton — Deep sequencing reveals important roles of microRNAs in response to drought and salinity stress in cotton — Supplementary Data 

# Deep sequencing reveals important roles of microRNAs in response to drought and salinity stress in cotton

## Supplementary Data

Data files

**Files in this Data Supplement:**

- Supplementary Data - Supplementary Data
